# Characterization of the complete chloroplast genome of *Melaleuca cajuputi* subsp. *cumingiana* (Myrtaceae)

**DOI:** 10.1080/23802359.2020.1871438

**Published:** 2021-02-09

**Authors:** Danhui Liang, Liming Zhu, Yuyu He, Xin Xiong

**Affiliations:** a The No. 15 Middle School of Yueyang City, Yueyang, China; b Key Laboratory of Vegetation Restoration and Management of Degraded Ecosystem, South China Botanical Garden, Chinese Academy of Sciences, Guangzhou, China

**Keywords:** *Melaleuca*, NOVOPlasty software, essential oil, traditional medicine

## Abstract

Plants in the genus *Melaleuca* have been widely used as traditional medicine mainly because of their broad spectrum antimicrobial activity. In this study, we reported the complete chloroplast genome of *Melaleuca cajuputi* subsp. *cumingiana*. The chloroplast genome of this species is 158,855 bp in length, including a pair of inverted repeat regions (IRs) (26,727 bp) that is divided by a large single-copy (LSC) area (87,338 bp) and a small single-copy (SSC) area (18,063 bp). The circular chloroplast genome of *M. cajuputi* subsp. *cumingiana* contains 135 unique genes, composing of 87 protein-coding genes, 40 tRNA genes, and eight rRNA genes. Phylogenetic analysis indicates that *M. cajuputi* subsp. *cumingiana* was clustered with species in the tribe Melaleuceae. This complete chloroplast genome of *M. cajuputi* subsp. *cumingiana* will provide a powerful tool to accelerate breeding, biotechnological and phylogenetic study.

Plants belonging to *Melaleuca* L. genus (Myrtaceae family) have been used as traditional medicine for many years (Sharifi-Rad et al. 2017), mainly because of their broad spectrum antimicrobial activity (Zhang et al. 2018). *Melaleuca cajuputi* Powell is a multi-purpose tree as its piles and frame poles provide construction materials, leaves produce essential oil, flowers attract honey bees (Doran and Turnbull 1997; Quat and Cuong 2005), and timbers can be used for pulp, fiber, and particle board (Trung 2008). In recent years, this plant was also developed for allelopathic herbicides instead of chemical herbicides (Kueh et al. 2019). There are three recognized subspecies: *cajuputi*, *cumingiana*, and *platyphylla* (Craven and Barlow 1997), which are native to Australia and adjacent areas such as Papua New Guinea, Indonesia, and Malaysia (Brophy et al. 2013). Breeding program of *M. cajuputi* subsp. *cajuputi* has mainly targeted essential oil yield (Kartikawati et al. 2015), while *M. cajuputi* subsp. *cumingiana* Barlow was widely planted for its wood production (Thiet et al. 2017; Nguyen et al. 2019). However, only a few genomic resources have been reported in this species (Beheregaray and Sunnucks 2000).

In higher plants, chloroplast genome is often used for phylogenetic analysis and domestication studies (Jansen et al. 2007). The whole chloroplast genome sequences also have demonstrated the potential to understand structure and functional evolution (Jansen et al. 2007; Moore et al. 2010). In genus *Melaleuca*, the chloroplast genome such as *M. alternifolia* and *M. rigidus* (which was initially described as a member of the genus *Callistemon*) has been reported (Liu et al. 2019, 2020), but the chloroplast genome of *M. cajuputi* has not been reported. Here, we sequenced and analyzed the complete chloroplast genome sequence of *M. cajuputi* subsp. *cumingiana* based on the Illumina sequencing data. The objective of this study was to characterize the complete chloroplast genome sequence of *M. cajuputi* as a resource for future genetic studies on this and other related species.

Voucher specimens of *M. cajuputi* subsp. *cumingiana* were collected from South China Botanical Garden, Chinese Academy of Sciences (Guangzhou, China; 113°21′7″E, 23°10′47″N), and deposited at the herbarium of South China Botanical Garden (accession number: SCBG-CF-2071). Total genomic DNA was extracted from fresh leaves using the CTAB-chloroform protocol (Doyle and Doyle 1987). The high-throughput sequencing (pair-end 150 bp) was performed on an Illumina XTen platform and it generated ∼3.07 Gb raw data. The cp genome was assembled by using the program NOVOPlasty (Dierckxsens et al. 2017). A ribulose-1, 5-bisphosphate carboxylase/oxygenase (*rbcL*) gene sequence from *M. alternifolia* (GenBank accession no. MN310606) was used as seed sequence, and the whole cp genome sequence of *M. alternifolia* and *Eucalyptus grandis* (GenBank accession no. NC_014570) was used as a reference to resolve the inverted repeat (IR) in the chloroplast genome of *M. cajuputi* subsp. *cumingiana*. The assembled chloroplast genome was annotated using PGA (Qu et al. 2019) and GeSeq (Tillich et al. 2017). For necessary genes, positions of start and stop codons and boundaries between exons and introns were manually corrected. The annotated chloroplast genomic sequence has been deposited in GenBank with an accession number: MT731621.

The complete chloroplast genome of *M. cajuputi* subsp. *cumingiana* is 158,855 bp in length, and has a typical quadripartite construction, which contains two inverted repeat regions (IRa and IRb) of 26,727 bp that is insulated by a large single-copy (LSC, 87,338 bp) and a small single-copy (SSC, 18,063 bp). The total GC content of complete chloroplast genome, LSC, SSC, IR regions is 36.8%, 55.0%, 11.4%, and 33.6%, respectively. The complete chloroplast genome of *M. cajuputi* subsp. *cumingiana* contains 135 unique genes, including 87 protein-coding genes, 40 tRNA genes, and eight rRNA genes. Introns are present in 19 of the annotated genes. Five of the intron containing genes contain three exons. Most of these genes are single-copy genes. However, 19 genes were duplicated in IR regions.

To confirm the phylogenetic position of *M. cajuputi* subsp. *cumingiana*, the complete chloroplast genomes of 16 published species within Myrtaceae and one outgroup (*Punica granatum*, Lythraceae, MK603512) were downloaded from the NCBI GenBank database. Ninety-four chloroplast genes shared by all species in this analysis were extracted, and were aligned by using MUSCLE (Edgar 2004). We concatenated these genes and then constructed a maximum-likelihood tree (Figure 1) using IQ-TREE (Nguyen et al. 2015). Phylogenetic analysis strongly supported that *M. cajuputi* subsp. *cumingiana* was closely related to species in tribe Melaleuceae (Figure 1), which is consistent with the previous study in Myrtaceae (Thornhill et al. 2015). In addition, phylogenetic position of *Melaleuca rigidus*, initially described as *Callistemon rigidus*, also supported the sinking of *Callistemon* into *Melaleuca* (Craven 2006). In conclusion, we assembled the first chloroplast genome of *M. cajuputi* and it will provide a solid foundation for phylogenetic and evolutionary studies in *Melaleuca* and is expected to contribute to improving *M. cajuputi* breeding.

**Figure 1.**
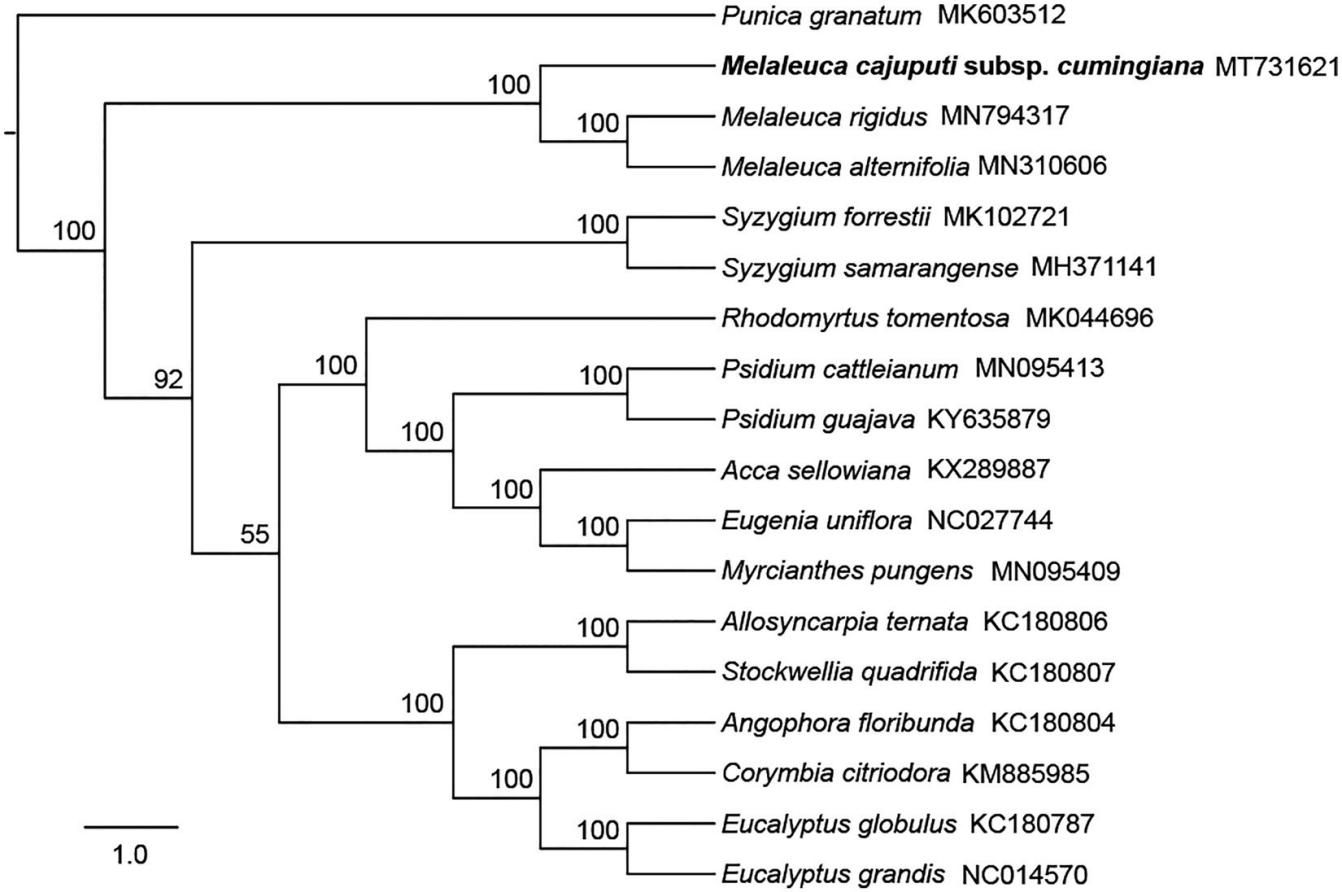
Maximum-likelihood tree shows the relationship among *M. cajuputi* subsp. *cumingiana* and other 16 species within Myrtaceae and one outgroup species (*Punica granatum*), using chloroplast gene sequences. Bootstrap supports based on 1000 replicates are given at the node.

## Data Availability

The contact person of specimen is Chen Feng (fengchen0215@scbg.ac.cn). The raw sequencing data of *M. cajuputi* subsp. *cumingiana* have been deposited in the NCBI Sequence Read Archive under accession numbers PRJNA674705. The chloroplast genome of the *M. cajuputi* subsp. *cumingiana* was submitted to GenBank under accession number: MT731621. Treefile of 18 species and genes for phylogenetic analysis were deposited at Figshare: https://doi.org/10.6084/m9.figshare.13194128.v1.
